# Autoimmune pancreatitis results from loss of TGFβ signalling in S100A4-positive dendritic cells

**DOI:** 10.1136/gut.2008.170779

**Published:** 2009-08-04

**Authors:** C S Boomershine, A Chamberlain, P Kendall, A-R Afshar-Sharif, H Huang, M K Washington, W E Lawson, J W Thomas, T S Blackwell, N A Bhowmick

**Affiliations:** 1 Department of Medicine, Vanderbilt-Ingram Cancer, Vanderbilt University, Nashville, Tennessee, USA; 2 Department of Urologic Surgery, Vanderbilt-Ingram Cancer, Vanderbilt University, Nashville, Tennessee, USA; 3 Department of Pathology, Vanderbilt-Ingram Cancer, Vanderbilt University, Nashville, Tennessee, USA

## Abstract

**Background and aims:**

Autoimmune pancreatitis (AIP) is a poorly understood human disease affecting the exocrine pancreas. The goal of the present study was to elucidate the pathogenic mechanisms underlying pancreatic autoimmunity in a murine disease model.

**Methods:**

A transgenic mouse with an S100A4/fibroblast-specific protein 1 (FSP1) Cre-mediated conditional knockout of the transforming growth factor β (TGFβ) type II receptor, termed Tgfbr2*^fspKO^*, was used to determine the direct role of TGFβ in S100A4^+^ cells. Immunohistochemical studies suggested that Tgfbr2*^fspKO^* mice develop mouse AIP (mAIP) characterised by interlobular ductal inflammatory infiltrates and pancreatic autoantibody production. Fluorescence-activated cell sorting (FACS)-isolated dendritic cells (DCs) from diseased pancreata were verified to have S100A4-Cre-mediated DNA recombination.

**Results:**

The Tgfbr2*^fspKO^* mice spontaneously developed mAIP by 6 weeks of age. DCs were confirmed to express S100A4, a previously reported protein expressed by fibroblasts. Adoptive transfer of bone marrow-derived DCs from Tgfbr2*^fspKO^* mice into 2-week-old syngenic wild-type C57BL/6 mice resulted in reproduction of pancreatitis within 6 weeks. Similar adoptive transfer of wild-type DCs had no effect on pancreas pathology of the host mice. The inability to induce pancreatitis by adoptive transfer of Tgfbr2*^fspKO^* DCs in adult mice suggested a developmental event in mAIP pathogenesis. Tgfbr2*^fspKO^* DCs undergo elevated maturation in response to antigen and increased activation of naïve CD4-positive T cells.

**Conclusion:**

The development of mAIP in the Tgfbr2*^fspKO^* mouse model illustrates the role of TGFβ in maintaining myeloid DC immune tolerance. The loss of immune tolerance in myeloid S100A4^+^ DCs can mediate mAIP and may explain some aspects of AIP disease pathogenesis.

Autoimmunity to pancreatic antigens can result in a number of human diseases including insulin-dependent diabetes mellitus (IDDM) and autoimmune pancreatitis (AIP). While the immune mechanisms underlying the development of IDDM are well known, information on the immunopathogenesis of AIP is limited.^1^ The specific pancreatic histopathology of the disease includes dense inflammatory infiltrates composed primarily of T cells and macrophages limited to interlobular ducts of the exocrine pancreas. However, AIP is one manifestation of immunoglobulin G4 (IgG4)-related sclerosing disease, a systemic disease characterised by periductal lymphocytes, plasma cells and macrophage infiltration of various organs including the pancreas, salivary gland, kidney, lung and prostate, with cellular and storiform fibrosis with progressive acinar atrophy and obliterative phlebitis.^2^ While the pathogenesis of AIP is poorly understood, it is associated with elevated Ig levels caused by the formation of autoantibodies against pancreatic antigens.^3^

Transforming growth factor β (TGFβ) is a pleiotropic cytokine that regulates immune function.^4^ TGFβ signalling requires binding to the TGFβ type II receptor (Tgfbr2) for downstream activity. We previously reported spontaneous development of forestomach squamous cell carcinoma and prostatic intraepithelial neoplasia in mice with fibroblastic conditional knockout of the *Tgfbr2* gene.^5^ These mice, termed Tgfbr2*^fspKO^*, were developed by crossing Tgfbr2*^floxE2/floxE2^* mice to transgenic mice expressing Cre recombinase driven by the FSP-1 (fibroblast specific protein-1 or S100A4) promoter. S100A4 expression was initially characterised in cells of mesenchymal origin including stromal fibroblasts and epithelial cells undergoing epithelial–mesenchymal transdifferentiation.^6^ S100A4 expression was subsequently identified in immunological tissues including spleen and lymph nodes.^7^ However, the identity and function of immune cells expressing the intracellular S100A4 protein have not been determined. Since TGFβ signalling requires the presence of Tgfbr2 on the cell surface,^8^ cells expressing S100A4 in Tgfbr2*^fspKO^* mice lack the ability to respond to TGFβ. Tgfbr2*^fspKO^* mice displayed slowed somatic growth beginning 2 weeks after birth and died by postnatal week 6.^5^ In studying the cause of premature death in Tgfbr2*^fspKO^* mice, we histologically identified pancreatitis. The data indicate the Tgfbr2*^fspKO^* mice develop a disease with the pancreatic manifestations of human AIP, and S100A4^+^ myeloid dendritic cells (DCs) lacking maturational regulation by TGFβ mediate the development of autoimmune disease.

## Materials and methods

### Animals

Mice were maintained and genotyped as previously described.^5^,^9^ All animals were backcrossed at least 13 generations onto the C57BL/6 strain (Harlan Laboratories. Indianapolis, Indiana, USA) and housed in sterile, pathogen-free conditions. The studies described have been reviewed and approved by the Vanderbilt Institutional Animal Care and Use Committee.

### Immunolocalisation and β-galactosidase activity

Pancreata were fixed in 4% paraformaldehyde and processed into paraffin blocks. H&E, Masson’s trichrome and immunohistochemical (IHC) staining were performed on 5 μm paraffin sections. IHC was performed by standard de-paraffinisation and rehydration steps followed by blocking with blocking buffer (10% fetal bovine serum (FBS) in phosphate-buffered saline (PBS)) for 1 h. Primary antibodies were incubated overnight at 4°C, followed by Envision-plus antimouse or antirabbit 3-diaminobenzidine detection kits (Dako, Carpinteria, California, USA). For determination of β-galactosidase activity, the pancreata were fixed in 4% paraformaldehyde for 1 h, washed, and incubated in X-gal for 4 h as previously described.^9^

For determination of pancreatic autoantibody expression, 10 μm frozen sections from canine pancreatic tissues were used to prevent non-specific staining seen with murine tissue. The sections were incubated in blocking buffer for 1 h and then in serum from either Tgfbr2*^fspKO^* or Tgfbr2*^floxE2/floxE2^* mice for 30 min (1:50 dilution in blocking buffer) prior to detection with antimouse IgG Fab labelled with Alexa Fluor 595 and Hoechst nuclear counterstain. Slides were visualised on a Nikon Axiophot epifluorescent microscope.

### Fluorescence-activated cell sorting (FACS)

Pancreas, spleen and bone marrow cells were analysed by FACS as previously described.^10^ Briefly, pancreata were harvested and injected with 1 mg/ml collagenase P (Roche Diagnostics, Nutley, New Jersey, USA) in Hank’s balanced salt solution (HBSS) followed by incubation in collagenase solution for 30 min at 37°C with agitation.^10^ After digestion, the tissue was drawn through an 18-gauge needle several times to dissociate the cells prior to FACS analysis.

Cells (1×10^6^ live cells) were blocked using anti-Fc antibody (Abcam, Cambridge, Massachusetts, USA) and stained in PBS containing 3% FBS and 2.5% sodium azide. 7-Actinomycin D (7-AAD) at 10 ng/ml (Sigma-Aldrich, St Louis, Missouri, USA) was added during antibody incubation to identify dead cells. Singly labelled cell aliquots along with cells stained with fluorochrome-labelled rat isotype control antibodies (BD Pharmingen, Franklin Lakes, New Jersey, USA) were used as compensation and negative staining controls, respectively. The Cytofix/Cytoperm Plus kit (BD Pharmingen) was used to identify intracellular S100A4 expression. Biotinylated S100A4 primary antibody (provided by Dr Eric Neilson, Vanderbilt University) was used with streptavidin-conjugated fluorescent dye. Labelled cells were analysed using a FACSCalibur flow cytometer (BD Biosciences). FACS data were analysed using WinMDI software (J Trotter, Scripps Institute, San Diego, California, USA). Numbers in each quadrant indicate mean percentage of cells (SD) from three replicate experiments performed using SPSS 16.0 (SPSS, Chicago, Illinois, USA).

CD11c-positive pancreatic cells were isolated by FACS (FACSAria, BD Biosciences) from Tgfbr2*^fspKO^* mice and Tgfbr2*^floxE2/floxE2^* littermate controls. Genomic DNA was isolated from cell aliquots using an Extract-N-Amp Tissue PCR kit (Sigma-Aldrich). PCR was conducted using sense (5′-AGGTGTAGAGAAGGCACTTAGC-3′) and antisense (5′-CTAATCGCCATCTTCCAGCAGG-3′) primers to identify cells that had undergone recombination of the *Tgfbr2* gene. DNA isolated from mouse ear clippings of Tgfbr2*^fspKO^* and Tgfbr2*^floxE2/floxE2^* mice were used as positive and negative controls, respectively.

### Generation of bone marrow-derived DCs

Myeloid DCs were generated as described by Lutz *et al*.^11^ Briefly, the flushed bone marrow cells, depleted of red blood cells, were plated in complete RPMI (containing 10% fetal calf serum, 55 μM 2-mercaptoethanol, 100 U/ml penicillin G sodium, 100 μg/ml streptomycin sulfate, 0.01 M HEPES and 0.08 M l-glutamine (Invitrogen, Grand Island, New York, USA)) with 20 ng/ml recombinant granulocyte–monocyte colony-stimulating factor (GM-CSF; PeproTech, Rocky Hill, New Jersey, USA). Following 10 days of culturing, non-adherent DCs for use in experiments were removed from cultures by vigorous pipetting and used for experiments after DC purity was insured by FACS.

### Generation of DC chimeric mice

Bone marrow-derived DCs from Tgfbr2*^fspKO^* mice and Tgfbr2*^floxE2/floxE2^* littermate controls were produced as above and washed twice in PBS prior to suspension in PBS at a concentration of 1×10^7^ cells/ml. Two independent experiments were conducted wherein 1×10^6^ bone marrow-derived DCs from either Tgfbr2*^fspKO^* or Tgfbr2*^floxE2/floxE2^* mice were injected into the peritoneal space of either 2-week-old or adult syngeneic C57BL/6 mice using an insulin syringe in 100 μl of PBS (n = 12). Pancreata from all mice were harvested 6 weeks later and examined histologically.

### DC maturation evaluation

Bone marrow-derived DCs from Tgfbr2*^fspKO^* and Tgfbr2*^floxE2/floxE2^* littermate control mice were generated as described above. DCs were treated overnight with 1 μg/ml lipopolysaccharide (LPS), 500 U/ml tumour necrosis factor α (TNFα) or 20 μg/ml zymosan to induce maturation. A total of 1×10^6^ treated and untreated DCs from each group underwent FACS as described above for major histocompatibility complex (MHC) class II, CD11c, CD86 (BD Pharmingen) and 7-AAD. The percentage of mature DCs was determined for each group and treatment as defined by cells simultaneously staining positive for CD11c and highly positive for CD86 and MHC class II after gating out dead (7-AAD-positive) cells. Experiments were conducted in triplicate with results reported as mean percentage mature DCs (SD), and one-way analysis of variance (ANOVA) was employed to compare Tgfbr2*^fspKO^* and Tgfbr2*^floxE2/floxE2^* DCs using SPSS 16.

### T cell proliferation assay

Spleens from OT-II.1 transgenic mice (Jackson Laboratories, Bar Harbor, Michigan, USA) with CD4^+^ T cell receptors specific for ovalbumin were harvested using aseptic technique, and single-cell suspensions were made by passing tissue through a nylon cell strainer. Red blood cells (RBCs) were lysed by Tris-ammonium chloride treatment, and CD4^+^ T cells were isolated using a magnetic-activated cell separation (MACS) column (Miltenyi Biotec, Auburn, Califonia, USA). T cell purity was confirmed by FACS of presort and postsort aliquots using fluorescence-conjugated antibodies specific for T cells (CD4), B cells (B220) and DCs (CD11c). Initially, bone marrow-derived DCs from Tgfbr2*^fspKO^* and Tgfbr2*^floxE2/floxE2^* control mice were incubated with ovalbumin (Sigma-Aldrich) at 100 μg/ml for 24 h prior to washing twice with PBS through centrifugation and resuspension, and irradiation with 10 Gy of ^127^Cs to prevent proliferation. Then ovalbumin-pulsed DCs were added to 100 000 OT-II.1 CD4^+^ T cells in triplicate to each well of a 96-well, flat-bottomed plate (Corning, Lowell, Massachusetts, USA) and incubated in complete RPMI. On the third day of treatment, [^3^H]thymidine incorporation assay was quantified by scintillation counting.^12^ Repeated-measures ANOVA was used to compare change in scintillation count with DC number between Tgfbr2*^fspKO^* and Tgfbr2*^floxE2/floxE2^* DCs using SPSS 16.0.

## Results

### The Tgfbr2*^fspKO^* mouse is a murine model of AIP (mAIP)

**Figure 1:**
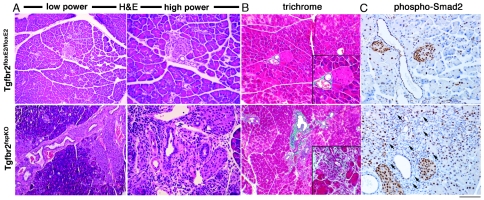
Tgfbr2*^fspKO^* mice develop pancreatitis. Representative views of H&E-stained pancreatic tissue from Tgfbr2**^floxE2/floxE2^** and Tgfbr2**^fspKO^** mice showing low and high power views (n = 20, scale bar represents 20 and 10 μm respectively). (B) Masson’s trichrome staining of collagen suggests minimal fibrotic reaction in the pancreatitis tissues of Tgfbr2**^fspKO^** mice (n = 6, scale bar represents 20 *μm). (C) Phosphorylated Smad-2 immunohistochemistry indicates loss of transforming growth factor β (TGFβ) signalling in the areas of pancreatitis in Tgfbr2*^fspKO^** mice (arrows; n = 6, scale bar represents 10 *μm).*

**Figure 2:**
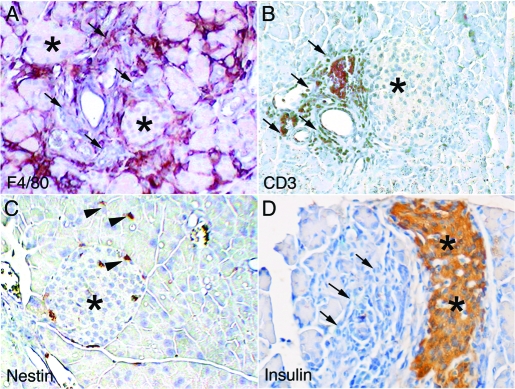
Pancreatitis in Tgfbr2*^fspKO^* mice resembles human autoimmune pancreatitis (AIP). Representative immunohistochemistry stains of pancreas from Tgfbr2*^fspKO^* mice using antibodies against (A) F4/80 (macrophage), (B) CD3 (T cells), (C) nestin (stellate cells) and (D) insulin (n = 6, scale bar represents 10 μm). Asterisks indicate islets and arrows indicate areas of pancreatitis. Arrowheads indicate nestin-positive cells.

**Figure 3:**
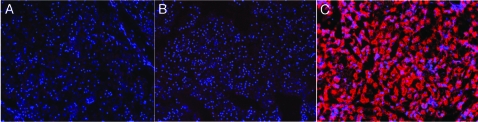
Tgfbr2*^fspKO^* mice produce pancreatic autoantibodies. Dog pancreas was incubated with (A) phosphate-buffered saline or serum from (B) Tgfbr2**^floxE2/floxE2^** (n = 4) and (C) Tgfbr2**^fspKO^** mice (n = 8). The immunoreactivity of the tissues was subsequently visualised with antimouse immunoglobulin G Fab fragment (in red) and Hoechst nuclear counterstain (in blue).

Histological sections from pancreata of Tgfbr2*^fspKO^* mice had periductal, interlobular inflammatory infiltrates that were not present in littermate control Tgfbr2*^floxE2/floxE2^* mice (fig 1). There was evidence of acinar metaplasia and expansion of fibroblastic cells periductally by 6 weeks of age without evidence for pancreatic islet inflammatory infiltration in these mice. The Tgfbr2*^fspKO^* pancreata had no gross pathological differences from the Tgfbr2*^floxE2/floxE2^* age-matched littermates. Masson’s trichrome staining suggested no significant fibrosis, as indicated by the lack of collagen deposition in either the Tgfbr2*^fspKO^* or Tgfbr2*^floxE2/floxE2^* pancreata. Immunolocalisation of phosphorylated Smad2, indicating active TGFβ signalling, was seen in pancreatic cells from both mice but was absent in inflammatory areas of acinar metaplasia and stromal expansion in Tgfbr2*^fspKO^* pancreata. As in human AIP, the inflammatory infiltrates were composed predominantly of macrophages and T cells with sparing of the endocrine pancreas (fig 2A and B, respectively). Further evidence for a non-fibrotic pancreatitis phenotype in Tgfbr2*^fspKO^* mice was demonstrated by the lack of expansion of nestin-positive stellate cells (fig 2C). As in human AIP, endocrine function did not seem to be overtly impaired in Tgfbr2*^fspKO^* mice, as determined by insulin (fig 2D) and somatostatin expression in the islets and serum (data not shown). Autoantibody production against pancreatic proteins is a hallmark of human AIP.^2^ To determine if Tgfbr2*^fspKO^* mice produced pancreatic autoantibodies, canine pancreatic tissue was incubated with serum from either Tgfbr2*^fspKO^* mice or Tgfbr2*^floxE2/floxE2^* littermate controls. Tgfbr2*^fspKO^* serum was found to contain mouse antibodies against pancreatic acinar tissue that were absent in serum from control, Tgfbr2*^floxE2/floxE2^* animals (fig 3).

**Figure 4:**
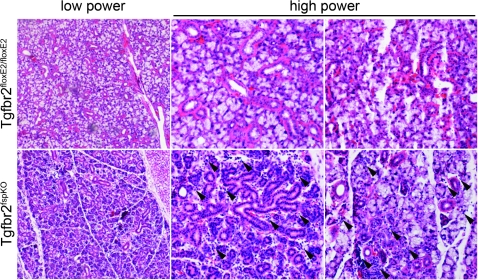
The salivary gland histology of Tgfbr2*^fspKO^* mice differs from that of Tgfbr2**^floxE2/floxE2^** mice. The widespread infiltration of inflammatory cells is appreciated in the low power field. Arrowheads indicate inflammatory cells in the high power fields (n = 6).

Human AIP can be characterised by a systemic manifestation of the autoimmune disease associated with extrapancreatic organ involvement. We reviewed H&E stains on lung, kidney, salivary gland and liver. Only the salivary gland of Tgfbr2*^fspKO^* mice differed from those of Tgfbr2*^floxE2/floxE2^* control mice. Figure 4 illustrates significant inflammatory infiltration in the salivary gland of Tgfbr2*^fspKO^* mice that was not present in Tgfbr2*^floxE2/floxE2^* mice. There was global involvement of inflammatory infiltrates associated with focal reactive metaplasia of the glandular epithelia. Both the pancreatic and salivary phenotype were observed only in Tgfbr2*^fspKO^* mice with 100% penetrance. Taken together, these results indicate that the Tgfbr2*^fspKO^* mouse is a spontaneous animal model for mAIP with parallels to human AIP at the histological level.

### Myeloid DCs express S100A4

**Figure 5:**
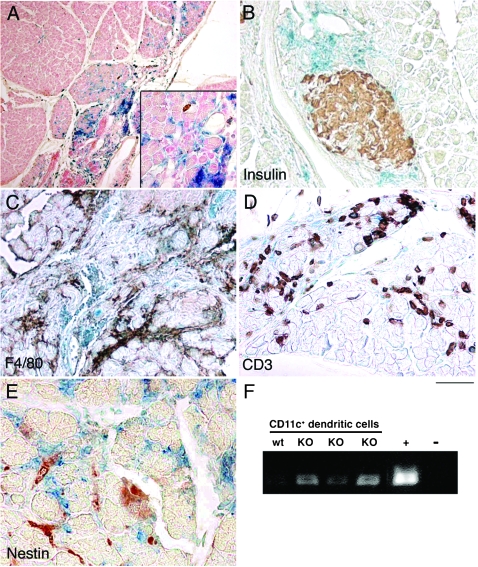
Dendritic cells (DCs) lacking the ability to respond to transforming growth factor β (TGFβ) are present in diseased pancreata. β-Galactosidase activity in conjunction with immunohistochemical stains of pancreas from Rosa26/Tgfbr2*^fspKO^* mice indicates fibroblast-specific protein (FSP)-Cre-mediated DNA recombination (A–E). (A) *β-Galactosidase activity (blue) is associated with pancreatitis with nuclear fast red counterstain (red). Concomitant β-galactosidase with immunostaining (brown) for (B) insulin, (C) F4/80 (for macrophages), (D) CD3 (for T cells) and (E) nestin (for stellate cells) did not suggest DNA recombination in these cell types. (F) PCR analysis for Cre-recombinase activity in flow-sorted CD11c-positive pancreatic cells from wild-type (wt) and Tgfbr2*^fspKO^* (KO) suggests specific recombination in DCs (n = 6). Mouse tail DNA from Tgfbr2**^fspKO^** and Tgfbr2**^floxE2/floxE2^** mice was used as a positive (+) and negative (−) control, respectively.*

To identify specific cell type(s) knocked out for *Tgfbr2* by S100A4-Cre, Tgfbr2*^fspKO^* mice were further crossed with Rosa26 mice. β-Galactosidase reporter activity of the pancreata from Rosa26/Tgfbr2*^fspKO^* mice illustrated the presence of cells positive for Cre-mediated DNA recombination (fig 5A). Positive staining was exclusively found in the exocrine pancreas (fig 5B), with the suggested *Tgfbr2*-knockout cells co-localised to infiltrating inflammatory cells in the pancreatic parenchyma. The lack of co-immunostaining for F4/80, CD3 and nestin with β-galactosidase activity suggested that *Tgfbr2*-knockout cells were not macrophages, T cells or stellate cells, respectively (fig 5C–E). Due to their morphology and the autoimmune nature of mAIP, we hypothesised that *Tgfbr2*-knockout cells in the pancreas could be myeloid DCs. Efforts to immunostain for the DC marker CD11c on paraffin sections were unsuccessful, so the presence of *Tgfbr2*-knockout DCs in disease pancreata was assessed by FACS of pancreatic single-cell suspensions for cells expressing CD11c on their surfaces. Cre-mediated *Tgfbr2* recombination was confirmed by PCR on the genomic DNA from the sorted pancreatic DCs (fig 5E). Thus, S100A4-positive DCs lacking TGFβ regulation were present in the pancreata of Tgfbr2*^fspKO^* mice.

### Myeloid S100A4^+^ DCs lacking TGFβ regulation induce pancreatitis

**Figure 6:**
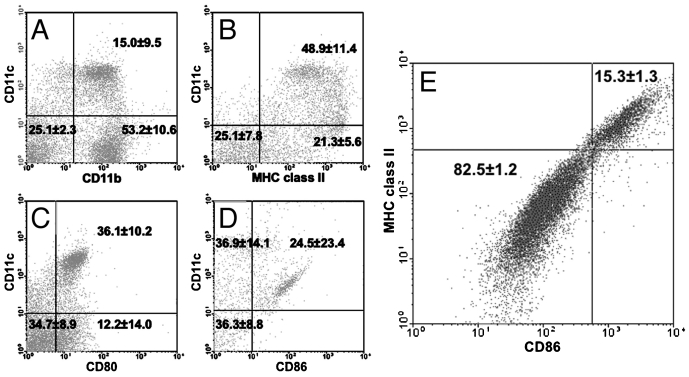
Splenic myeloid dendritic cells (DCs) express S100A4. Green fluorescent protein (GFP)-positive splenocytes from fibroblast-specific protein 1 (FSP1)·GFP mice simultaneously express CD11c and (A) CD11b, (B) major histocompatibilty comple (MHC) class II, (C) CD80 and (D) CD86. (E) GFP and CD11c double-positive bone marrow-derived DCs from FSP1·GFP mice demonstrate high positivity for MHC class II and CD86 expression (numbers in each quadrant indicate the percentage of viable cells ±SD) (n = 6).

**Figure 7:**
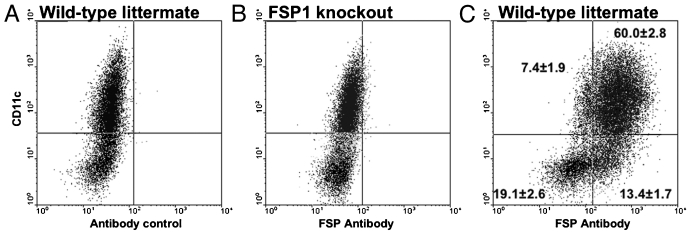
Bone marrow-derived dendritic cells (DCs) express S100A4. S100A4 expression by bone marrow-derived DCs were assessed by fluorescence-activated cell sorting (FACS) for CD11c with (A) isotype control antibody on wild-type DCs and (B) S100A4 antibody on S100A4 knockout mice. (C) DCs from wild-type mice were double-positive for CD11c and S100A4 expression (numbers indicate the percentage of live cells in each quadrant ± SD) (n = 6).

To characterise further immune cells expressing S100A4 that could be responsible for inducing mAIP, we conducted FACS experiments on splenocytes from FSP1·GFP (green fluoresent protein) transgenic mice.^13^ FSP1·GFP mice express GFP under control of the S100A4 promoter,^13^ and characterisation of GFP-positive splenocytes would allow for identification of candidate immune cells. A population of GFP-positive cells was found to co-express the myeloid DC markers CD11b and CD11c (fig 6A). Further, we identified splenocytes positive for both CD11c and GFP that expressed MHC class II and the co-stimulatory molecules CD80 and CD86 (fig 6B–D, respectively). We found no evidence for S100A4 expression by T cells, B cells or macrophages using antibodies against the cell-specific markers CD4 and CD8, B220 and F4/80, respectively (data not shown). To identify DCs definitively, CD11c-positive cells must be identified that simultaneous express high levels of MHC class II and co-stimulatory molecules, as this is pathognomonic for mature DCs.^14^ However, since DCs make up only 2–3% of total splenocytes, and only a small percentage of these DCs are mature, confirmatory evidence for S100A4 expression by DCs using FSP1·GFP splenocytes was limited. To overcome the problem of low numbers of splenic mature DCs, we generated myeloid DCs from the bone marrow of FSP1·GFP mice in vitro by treatment with GM-CSF.^11^ Bone marrow-derived S100A4-positive myeloid DCs simultaneously expressed GFP, CD11c and a high level of both MHC class II and CD86 (fig 6E). Intracellular staining of similar bone marrow-derived DCs with an antibody specific for S100A4 provided independent confirmation for S100A4 expression by myeloid DCs (fig 7). The specificity of the S100A4 antibody was confirmed by the observed lack of staining in bone marrow-derived DCs from previously characterised FSP1 knockout mice (fig 7B).^13^

**Figure 8:**
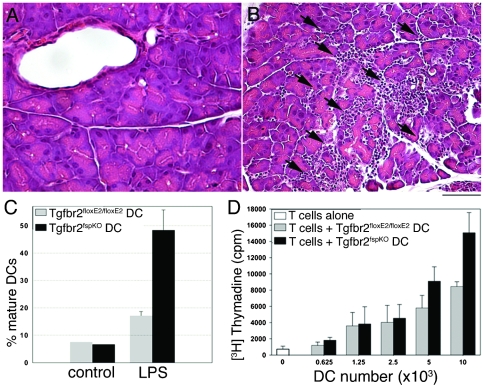
Tgfbr2*^fspKO^* dendritic cells (DCs) can induce autoimmune pancreatitis. Representative H&E-stained pancreatic tissue from 2-week-old wild-type mice after transfer of bone marrow-derived DCs from (A) Tgfbr2*^floxE2/floxE2^* control or (B) Tgfbr2*^fspKO^* mice (n = 12). Arrows indicate areas of inflammation. (C) Fluorescence-activated cell sorting (FACS) analysis of 1×10^6^ live bone marrow-derived DCs from Tgfbr2*^fspKO^* and Tgfbr2*^floxE2/floxE2^* mice before and after treatment with lipopolysaccharide (LPS) for 24 h to determine the percentage of mature DCs defined as live DCs simultaneously expressing CD11c and high levels of CD86 and major histocompatibility complex (MHC) class II. No significant difference was seen between untreated DCs, but LPS stimulation induced a significantly higher percentage of mature DCs in Tgfbr2*^fspKO^* cultures (p = 0.002). (D) Proliferation of OTII.1 CD4^+^ T cells stimulated with ovalbumin-pulsed bone marrow-derived DCs from Tgfbr2*^fspKO^* and Tgfbr2*^floxE2/floxE2^* mice. Tgfbr2*^fspKO^* DCs induced significantly higher T cell proliferation with increasing DC number (p = 0.05, untreated T cell control 335 (119) cpm).

To implicate S100A4^+^ DCs directly in the pathogenesis of mAIP, bone marrow-derived DCs generated from either Tgfbr2*^fspKO^* or Tgfbr2*^floxE2/floxE2^* control mice were transferred by intraperitoneal injection to two groups of 2-week-old syngeneic, wild-type mice. After 6 weeks, pancreata from all mice were harvested and examined for evidence of inflammation. Histologically, all mice receiving Tgfbr2*^fspKO^* DCs showed pancreatic inflammatory infiltrates, but mice receiving Tgfbr2*^floxE2/floxE2^* DCs failed to develop pancreatitis (fig 8A,B). Development of pancreatitis was age dependent, as adult mice receiving either Tgfbr2*^fspKO^* or Tgfbr2*^floxE2/floxE2^* DCs failed to develop pancreatitis under similar conditions (data not shown). Development of pancreatitis in Tgfbr2*^fspKO^* DC chimeras indicated that TGFβ signalling in S100A4-positive myeloid DCs is required to prevent development of mAIP and supports a role for TGFβ signalling in DC-mediated immune tolerance. The finding that development of pancreatitis was age dependent implicated an early pancreatic developmental event in mAIP pathogenesis.

### Tgfbr2*^fspKO^* DCs have altered maturation resulting in increased T cell activation

Since TGFβ is known to regulate DC function and control of DC maturation is needed to maintain immune tolerance, we hypothesised that Tgfbr2*^fspKO^* DCs lack normal maturational regulation, resulting in the development of autoimmunity. To study DC maturation, bone marrow-derived DCs from Tgfbr2*^fspKO^* and Tgfbr2*^floxE2/floxE2^* control mice were treated in vitro with molecules that induce DC maturation, including LPS, TNFα and zymosan. After 24 h, DCs were harvested and upregulation of the maturation markers MHC class II and CD86 was compared using FACS. While the number of mature DCs in untreated Tgfbr2*^floxE2/floxE2^* and Tgfbr2*^fspKO^* cultures was similar, LPS induced maturation of Tgfbr2*^fspKO^* DC cultures more than threefold compared with wild type DC cultures (fig 8C). Similar results were seen with TNFα and zymosan treatment (data not shown).

Upregulation of MHC class II and CD86 is necessary for induction of immunity through activation of naïve T cells.^15^,^16^ We hypothesised that mAIP occurs in Tgfbr2*^fspKO^* mice through overactivation of T cells by Tgfbr2*^fspKO^* DCs. To test this hypothesis, T cell proliferation assays were performed using ovalbumin-stimulated bone marrow-derived DCs from Tgfbr2*^fspKO^* and Tgfbr2*^floxE2/floxE2^* control mice and naïve splenic CD4^+^ T cells from syngeneic, transgenic OT-II.1 mice that express a single T cell receptor specific for ovalbumin.^17^ DCs from Tgfbr2*^fspKO^* mice induced significantly more T cell proliferation after exposure to ovalbumin than control DCs (fig 8D). This evidence implicated increased T cell activation due to the lack of TGFβ signalling in S100A4^+^ DCs as a mechanism for loss of immune tolerance to pancreatic antigens that results in the development of mAIP.

## Discussion

The present study provides evidence that TGFβ regulation of DCs mediates tolerance during pancreatic differentiation to prevent development of pancreatic autoimmunity. The Tgfbr2*^fspKO^* mouse models some histological aspects of human AIP, as Tgfbr2*^fspKO^* mice spontaneously develop interlobular inflammatory infiltrates of the exocrine pancreas associated with pancreatic autoantibody production. Myeloid DCs from both in vivo and in vitro sources were found to express S100A4. S100A4^+^ DCs were implicated in the pathogenesis of AIP by demonstrating the presence of DCs that had undergone Cre-mediated *Tgfbr2* recombination in pancreata from Tgfbr2*^fspKO^* mice, and by induction of pancreatitis in wild-type mice through transfer of DCs from Tgfbr2*^fspKO^* mice. The induction of pancreatitis by administration of Tgfbr2*^fspKO^* DCs to wild-type mice and increased Tgfbr2*^fspKO^* DC activation of naïve T cells provided direct evidence for the role of TGFβ in maintaining DC immune tolerance.

Although TGFβ is known to modulate DC maturity,^18^ our studies implicate disruption of TGFβ signalling in DCs as a critical event in the development of autoimmunity. DCs normally maintain immune tolerance by remaining immature during the presentation of self-antigens to T cells, resulting in T cell anergy. Since Tgfbr2*^fspKO^* DCs have increased maturation in response to activation, we hypothesise that autoimmunity occurs in the Tgfbr2*^fspKO^* mouse due to precocious DC maturation in response to self-antigen, resulting in the activation of self-reactive T cells. Studies using targeted disruption of *Tgfbr2* in helper T cells to induce autoimmunity are consistent with the idea that TGFβ affects autoimmunity by modulating interactions between DCs and T cells. By expressing a dominant-negative form of *Tgfbr2* under direction of the CD4 promoter, murine models of human primary biliary cirrhosis^19^ and inflammatory bowel disease^20^ associated with autoantibody production have been generated. In the model of inflammatory bowel disease, T cells were shown to evade immune tolerance by undergoing DC-independent activation.

Our model of mAIP differs from previous DC transfer models of autoimmune disease in that neither priming nor maturation of DCs was required prior to transfer to induce autoimmunity. However, our ability to induce autoimmunity was dependent on the age of the recipient mouse. Histological evidence of pancreatitis was only seen when Tgfbr2*^fspKO^* DCs were transferred to 2-week-old mice; transfer of Tgfbr2*^fspKO^* DCs to adult mice did not induce pancreatitis. Similarly, Tgfbr2*^fspKO^* mice had no gross phenotypic abnormalities or pancreatic inflammation by histology until after 2 weeks of age. Interestingly, the second postnatal week has previously been identified as a critical time for the development of pancreatic autoimmunity in the form of IDDM.^21^ During this time, the pancreas undergoes a developmental rearrangement that results in a wave of programmed cell death involving both exocrine and endocrine pancreatic tissue (personal communication).^22^ So, it remains unclear why Tgfbr2*^fspKO^* mice develop autoimmunity of the exocrine but not the endocrine pancreas. However, non-obese diabetic (NOD) mice are known to have increased cell death within the endocrine pancreas compared with normal mice.^23^ It is possible that the digestive enzymes present within pancreatic acinar cells may provide for a more pronounced inflammatory environment compared with the endocrine pancreas. Alternatively, by using the S100A4 promoter to disrupt TGFβ signalling, we have targeted a unique DC population with specific functional properties or a specific stage of DC maturation. Antigen-presenting cells, including DCs, recruited to remove the resulting cellular debris of the developmental rearrangement are exposed to large amounts of self-antigens, and abnormal DC maturation in response to antigens can lead to autoimmunity.^24^ It is likely that mAIP results in Tgfbr2*^fspKO^* mice through a similar mechanism involving abnormal DC maturation in response to exocrine pancreatic antigens.

Consistent with our results, lack of TGFβ regulation has previously been implicated in histological changes associated with mAIP.^25^ Mice overexpressing a dominant-negative form of *Tgfbr2* directed by the *pS2* mouse trefoil peptide promoter to target pancreatic acinar cells (pS2-dnRII) developed mAIP after cerulein treatment. Cerulein is a cholecystokinin analogue that induces cell death in exocrine pancreatic tissue at supraphysiological doses. PS2-dnRII mice do not spontaneously develop mAIP, and it is likely that cerulein treatment in combination with the loss of TGFβ signalling is required to induce an inflammatory milieu sufficient to overcome the normal DC tolerogenic mechanisms present in pS2-dnRII mice. Upregulation of MHC class II in pancreatic acini expressing dnRII was associated with mAIP in the PS2-dnRII mice. Analogously, the mAIP observed in Tgfbr2*^fspKO^* mice was associated with increased MHC class II expression in the Tgfbr2*^fspKO^* DCs, supporting TGFβ regulation of MHC class II expression in maintaining immune tolerance. The upregulation of MHC class II alone in PS2-dnRII was not sufficient for pathogenesis of mAIP, as cerulein treatment was required. This is probably due to the inflammatory milieu induced by cerulein that is necessary for the production of co-stimulatory signals needed for T cell activation that are not produced by pancreatic acini. mAIP spontaneously develops in Tgfbr2*^fspKO^* mice because DCs also upregulate co-stimulatory molecule expression (eg, CD86) along with MHC class II. Since Tgfbr2*^fspKO^* mice lack normal DC regulation of both MHC class II and co-stimulatory molecules, the antigens released due to cell death resulting from the pancreatic developmental rearrangement in Tgfbr2*^fspKO^* mice, or 2-week-old wild-type mice receiving Tgfbr2*^fspKO^* DCs, is sufficient to induce pancreatitis in these mice. This would explain why adult wild-type mice receiving Tgfbr2*^fspKO^* DCs do not develop pancreatitis, as the required inciting developmental event has passed. If true, cerulein treatment of adult wild-type mice receiving Tgfbr2*^fspKO^* DCs may induce mAIP. The spontaneous development of mAIP in Tgfbr2*^fspKO^* mice is probably a combinatorial effect of a developmentally induced apoptotic wave and altered regulation of Tgfbr2*^fspKO^* DC tolerance.

Our finding that loss of immune tolerance in myeloid S100A4^+^ DCs can mediate mAIP in mice may help to explain some pathogenic aspects of human AIP, an IgG4-related sclerosing disease. However, differences exist between the mAIP disease model and the human condition. The lack of pancreatic fibrosis in diseased Tgfbr2*^fspKO^* pancreata is likely to be due to the inability of fibroblasts in the transgenic mouse to respond to TGFβ, a potent mediator of collagen expression.^26^ The development of mAIP occurs in mice of both genders early in life, whereas human AIP typically affects older males.^2^ It also remains unclear why our Tgfbr2*^fspKO^* mice have involvement of some of the organs seen in IgG4-related sclerosing disease, including pancreas and salivary gland involvement, but not other organs such as lung or kidney. One explanation may lie in the fact that mice do not express the IgG4 subclass.^25^ We are hopeful that further work utilising our murine model will lead to a better understanding of disease pathogenesis in AIP.
